# Diagnosing and Managing Babesiosis: A Case Study of Timely Intervention in a Tick-Borne Illness

**DOI:** 10.7759/cureus.79658

**Published:** 2025-02-25

**Authors:** Anna Haymov, Sarmad Baloch, Cassie R Girardin

**Affiliations:** 1 Neurology and Neurosurgery, Lake Erie College of Osteopathic Medicine, Elmira, USA; 2 Hospital Medicine, Olean General Hospital, Olean, USA; 3 Neurology, Lake Erie College of Osteopathic Medicine, Elmira, USA

**Keywords:** a case study, babesia species, babesiosis, infectious disease medicine, tick-borne infections, timely intervention, treatment

## Abstract

Babesiosis is a tick-borne parasitic infection caused by the Babesia species and is primarily transmitted by deer ticks. The disease has become more prevalent due to climate change and expanding tick populations, making early diagnosis and intervention crucial. This case study details the clinical presentation, diagnostic challenges, and treatment of a 52-year-old female patient with babesiosis, emphasizing the importance of timely detection in tick-endemic regions. The patient presented with fever, chills, body ache, thrombocytopenia, and a history of tick exposure. Initial treatment for suspected sepsis was modified after consultation with infectious disease specialists, leading to the identification of Babesia on the blood smear and confirmation of the diagnosis. The patient was successfully treated with atovaquone and azithromycin, and her condition improved following appropriate management. This case highlights the need for heightened clinical suspicion in patients with febrile illnesses and tick exposure, as well as the value of early diagnostic testing and targeted therapy in improving patient outcomes.

## Introduction

Babesiosis is a tick-borne parasitic infection caused by the Babesia species and is primarily transmitted by *Ixodes scapularis* (deer tick). Once limited to the northeastern and north-central U.S., the geographic range of babesiosis is expanding due to climate change and tick migration, now affecting the Midwest, West Coast, and parts of Canada. The disease is increasingly recognized as a public health concern, particularly in areas where it was previously rare. It can also be transmitted through blood transfusions and organ transplants, which complicates its management. It poses a significant risk to immunocompromised individuals, the elderly, and those who have had a splenectomy or suffer from functional asplenism due to various conditions such as thalassemia and sickle cell disease [1,2].

The epidemiology of babesiosis has changed significantly in recent decades, with cases now being reported in urban and suburban areas due to the migration of infected ticks. Historically common in rural, tick-endemic regions with large deer populations, babesiosis has notably spread to areas such as the northeastern U.S., Long Island, the upper Midwest, and parts of New England. Between 2011 and 2015, babesiosis cases nearly doubled in Massachusetts and Rhode Island, with similar increases in Connecticut as ticks spread into new areas. From 2011 to 2019, the CDC reported a more than threefold rise in U.S. cases, particularly in Massachusetts, New York, and Connecticut [3,4]. Climate change is driving the expansion of tick populations into previously unaffected regions, a trend not limited to the U.S. Canada has also seen rising cases, indicating the global spread of the disease [4,5].

Diagnosing babesiosis remains challenging due to the nonspecific nature of its symptoms. The disease often presents with fever, fatigue, muscle aches, and chills, which overlap with other common febrile illnesses. These overlapping symptoms complicate the differentiation of babesiosis from other diseases, particularly in endemic areas where multiple tick-borne illnesses such as Lyme disease and anaplasmosis are prevalent, increasing the risk of coinfection. Diagnosis is primarily based on laboratory findings, including blood smears, polymerase chain reaction (PCR) testing, and serological assays [6]. Treatment typically involves atovaquone with azithromycin for mild cases, and clindamycin with quinine for severe cases. Prompt treatment is essential to prevent complications like hemolytic anemia and organ failure [7,8].

This case study highlights the clinical, diagnostic, and treatment challenges of babesiosis, emphasizing the importance of early detection, especially in areas where ticks are spreading. Enhanced public health surveillance and education on the risks of tick-borne diseases are critical to controlling the rise of babesiosis in newly affected regions.

## Case presentation

A 52-year-old female patient with a medical history of insulin-dependent diabetes mellitus, hypertension, hypercholesterolemia, and depression presented to the Emergency Department with fever, chills, body aches, malaise, and poor oral intake for the past week. She reported that her father, hospitalized in the same facility, has similar symptoms, and her husband had removed three ticks from her in the past two weeks. A prior Lyme disease test was negative. Her review of systems was notable for fever, headache, and generalized body aches, but she denied chest pain, shortness of breath, gastrointestinal symptoms, trauma, or weakness.

Physical examination revealed an ill-appearing but not acutely distressed patient, with stable vital signs except for a fever of 102.7°F, mild tachycardia, and mild respiratory rate elevation. Initial laboratory findings included thrombocytopenia, elevated liver enzymes, and hyperglycemia (Table 1).

**Table 1:** Complete blood count and chemistry WBC = White blood cells, RBC = Red blood cells, ESR = Erythrocyte sedimentation rate, BUN = Blood urea nitrogen, AST = Aspartate aminotransferase, ALT = Alanine aminotransferase

	Normal range	Day 1	Day 2	Day 3	Day 4
Hematology					
WBC (K/mm^3^)	4-10.5	4.8	2.8	3.6	4.3
Neutrophils (%)	40-74	72.4	69.1	70.3	72.3
Lymphocytes (%)	19-48	8.1	14.2	12.5	9.6
Monocytes (%)	3-9	19.2	14.2	15.2	16.5
Eosinophils (%)	0-7	0.3	1.9	1.3	0.7
Basophils (%)	0-2	0	0.6	0.7	0.9
RBC (M/µL)	4.2-5.4	3.6	3.36	3.3	2.87
Hemoglobin (gm/dL)	12.5-16.0	11	10.3	10.1	8.7
Hematocrit (%)	37-47	33.9	30.6	30.3	26.1
Mean Corpuscular Volume (fL)	78-100	94.1	90.9	91.8	90.8
Platelets (K/mm^3^)	150-450	37	20	20	43
Giant platelets	Absent	Present	-	-	-
ESR (mm/hr)	0-33	73	-	-	-
Haptoglobin (mg/dL)	43-212	<10	-	-	-
Chemistry					
Sodium (mmol/L)	136-145	126	137	138	135
Potassium (mmol/L)	3.5-5.1	4.7	3.5	3.9	3.8
Chloride (mmol/L)	98-110	97	109	111	109
Carbon dioxide (mmol/L)	20-31	23.6	22.3	22.2	19
BUN (mg/dL)	9-23	21	19	19	18
Creatinine (mg/dL)	0.55-1.02	1.38	0.99	1.04	1.02
Glomerular filtration rate (mL/min)	60-999	42.7	62.6	59.1	60.5
Glucose (mg/dL)	74-106	405	139	146	109
Lactic acid (mmol/L)	0.5-2.0	1.5	1.2	1.1	
Calcium (mg/dL)	8.7-10.4	8.3	7.9	7.7	7.5
Ferritin (ng/mol)	10-291	>1500	-	-	-
Total bilirubin (mg/dL)	0.2-1.2	1.3	1.0	-	-
Direct bilirubin (mg/dL)	0.0-0.3	0.5	-	-	-
Indirect bilirubin (mg/dL)	0.1-0.8	0.8	-	-	-
AST (U/L)	0-34	115	207	-	-
ALT (U/L)	10-49	85	141	-	-
Alkaline phosphatase (U/L)	46-116	105	147	-	-
Total protein (g/dL)	5.7-8.2	6.7	5.6	-	-
Albumin (g/dL)	3.4-5.0	2.7	2.1	-	-

Procalcitonin was also elevated (Table 2). Urinalysis did not indicate a urinary tract infection (UTI) but had significant levels of ketones and glucose.

**Table 2:** Additional tests ANA = Antinuclear antibody.

	Normal range	Test result
Lactate dehydrogenase (U/L)	120-246	455
C-Reactive Protein (mg/L)	0-3.01	185.94
Alpha 1 globulin (g/dL)	0.2-0.3	0.5
Alpha 2 globulin (g/dL)	0.5-0.9	0.5
Beta 1 globulin (g/dL)	0.4-0.6	0.4
Beta 2 globulin (g/dL)	0.2-0.5	0.5
Gamma globulin (g/dL)	0.8-1.7	1.3
Serum monoclonal protein (g/dL)	None	0.3
Triglycerides (mg/dL)	0-150	207
Cholesterol (mg/dL)	0-200	74
Lipase (U/L)	12-53	18
Vitamin B12 (pg/mL)	211-911	>2000
Folate (ng/mL)	5.38-24.00	17
Procalcitonin (ng/mL)	0.04-0.50	11.78
Acetone	Negative	Negative
ANA	Negative	Negative

**Figure 1:**
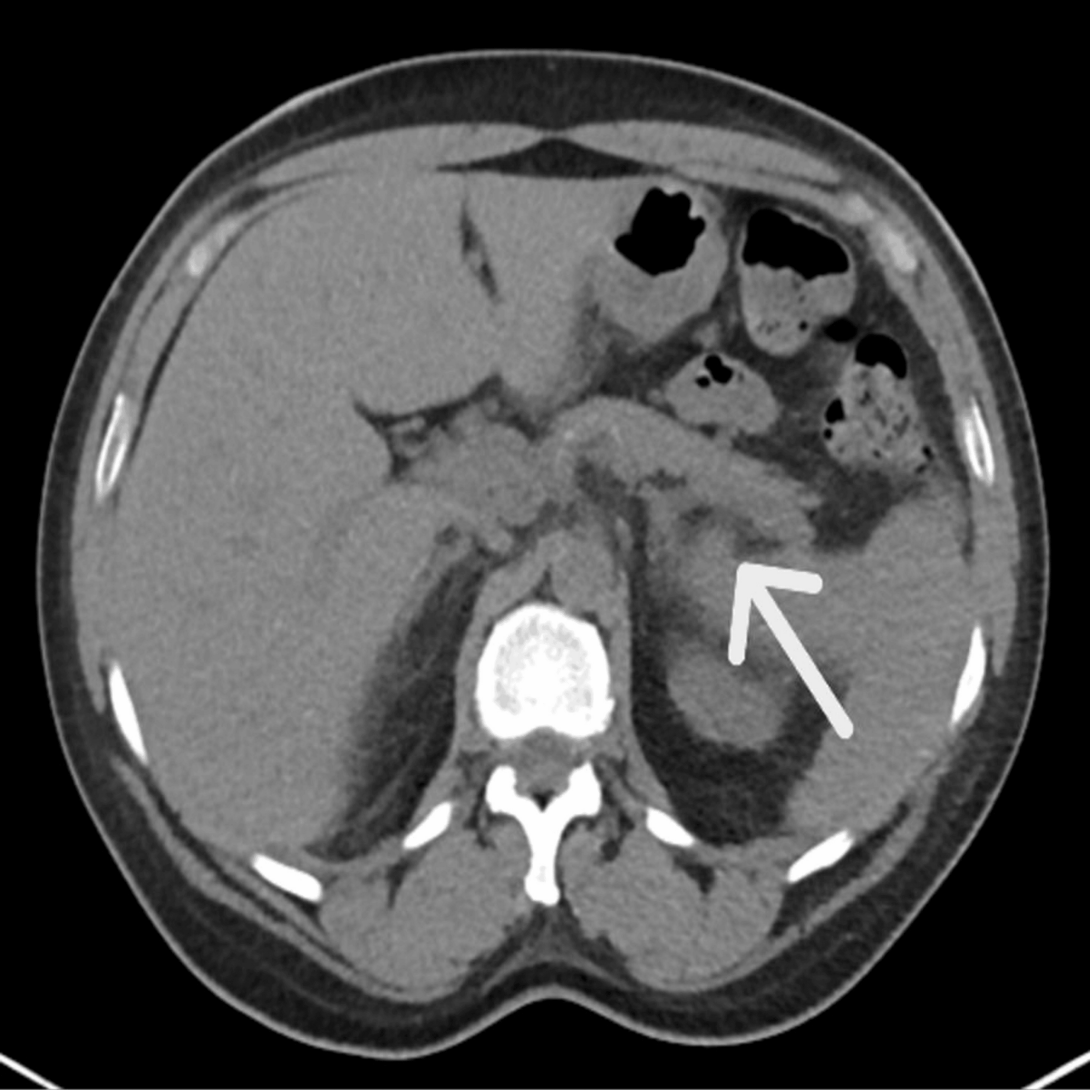
CT without contrast transverse view In this image, the borders of the pancreas are blurry, suggesting pancreatitis (white arrow). Other organs such as the liver and spleen, also evident here, are unremarkable.

Imaging including chest X-ray (CXR) showed no consolidation, while CT abdomen was concerning for acute pancreatitis (Figure 1).

However, acute pancreatitis was ruled out due to unremarkable lipase levels, suggesting CT findings were incidental. Blood cultures, HIV, hepatitis, and other infectious serologies were negative other than evidence of past Epstein-Barr infection confirmed with pathology a few days later (Table 3).

**Table 3:** Serology and microbiology EBV = Epstein-Barr virus, MRSA = Methicillin-resistant Staphylococcus aureus.

	Normal range	Test results
Lyme IgG	Negative	Negative
Lyme IgM	Negative	Negative
EBV nuclear antigen Ab (U/mL)	<18	263
EBV Capsid Ag IgG Ab (U/mL)	<18	>750
Hepatitis A IgM Ab	Nonreactive	Nonreactive
Hep Bs Ag	Nonreactive	Nonreactive
Hep C antibody	Nonreactive	Nonreactive
HIV 1 & 2 antibody	Nonreactive	Nonreactive
Influenza type A&B Ag	Negative	Negative
Rickettsia IgG and IgM antibody	Not detected	Not detected
SARS-Cov RT-PCR	Not detected	Not detected
Malaria smear	Negative	Organisms resembling Babesia present
EBV pathology	Negative	Suggestive of past EBV infection
Blood smear report	Negative	Intracellular organisms resembling Babesia species; >5% parasitemia
Stool cultures	E. Coli, Salmonella, Shigella and Campylobacter negative	E. coli, Salmonella, Shigella and Campylobacter negative
Nasopharyngeal swab	MRSA not detected	MRSA not detected
Blood cultures	No growth	No growth

The patient was initially started on cefepime and vancomycin for suspected sepsis, and intravenous fluids were administered. The working diagnosis included sepsis, possibly from a tick-borne illness such as Lyme disease, anaplasmosis or babesiosis, with associated thrombocytopenia and elevated liver enzymes. After an infectious disease consult, vancomycin was stopped, and doxycycline was continued. The blood smear originally meant to assess malaria showed the Maltese cross sign, indicating the presence of Babesia, and was confirmed with pathology a few days later. Atovaquone 750 mg twice daily and azithromycin 500 mg daily for 10 days were initiated for babesiosis. The patient’s thrombocytopenia was monitored, and no platelet transfusion was needed per the oncology consultation. Liver enzymes were expected to improve with treatment, and blood glucose levels were managed with insulin. Hypertension and hyperlipidemia were stable with home medications, and depression was managed with escitalopram. The patient was also monitored for hydration and hyponatremia, which improved with continued IV fluids.

Infectious disease continued to follow her case, and a transfer to a higher level of care for further evaluation of suspected parasitemia and potential plasmapheresis was recommended. The patient’s course remained stable and she was transferred to a tertiary care facility, with a resolution of the fever and an improvement in her condition over the following days.

## Discussion

Babesiosis, caused by *Babesia microti*, is mainly transmitted through infected *Ixodes scapularis *ticks but can also spread via blood transfusions, organ transplants, and rarely congenital transmission. High-risk groups include the elderly, immunocompromised individuals, and those without a spleen [1,2]. U.S. cases tripled from 2011 to 2019, spreading the disease from rural areas in the Northeast and Midwest to urban regions. This increase is partly due to climate change, which has expanded tick populations [3-5]. Understanding babesiosis better is crucial for improving prevention, diagnosis, and treatment as cases rise.

The clinical presentation of babesiosis is highly variable. While many infections are asymptomatic or cause only mild symptoms, others can lead to severe, life-threatening conditions, especially in high-risk groups. Patients often present with fever, chills, fatigue, body aches, and signs of hemolytic anemia, such as jaundice and thrombocytopenia. Severe cases may result in organ failure, including splenic rupture, and can even be fatal if not promptly treated. The increased frequency of severe cases in older adults and immunocompromised patients highlights the critical need for early detection and treatment and it is one of about 120 diseases that are nationally notifiable in the U.S. [3,4].

**Figure 2:**
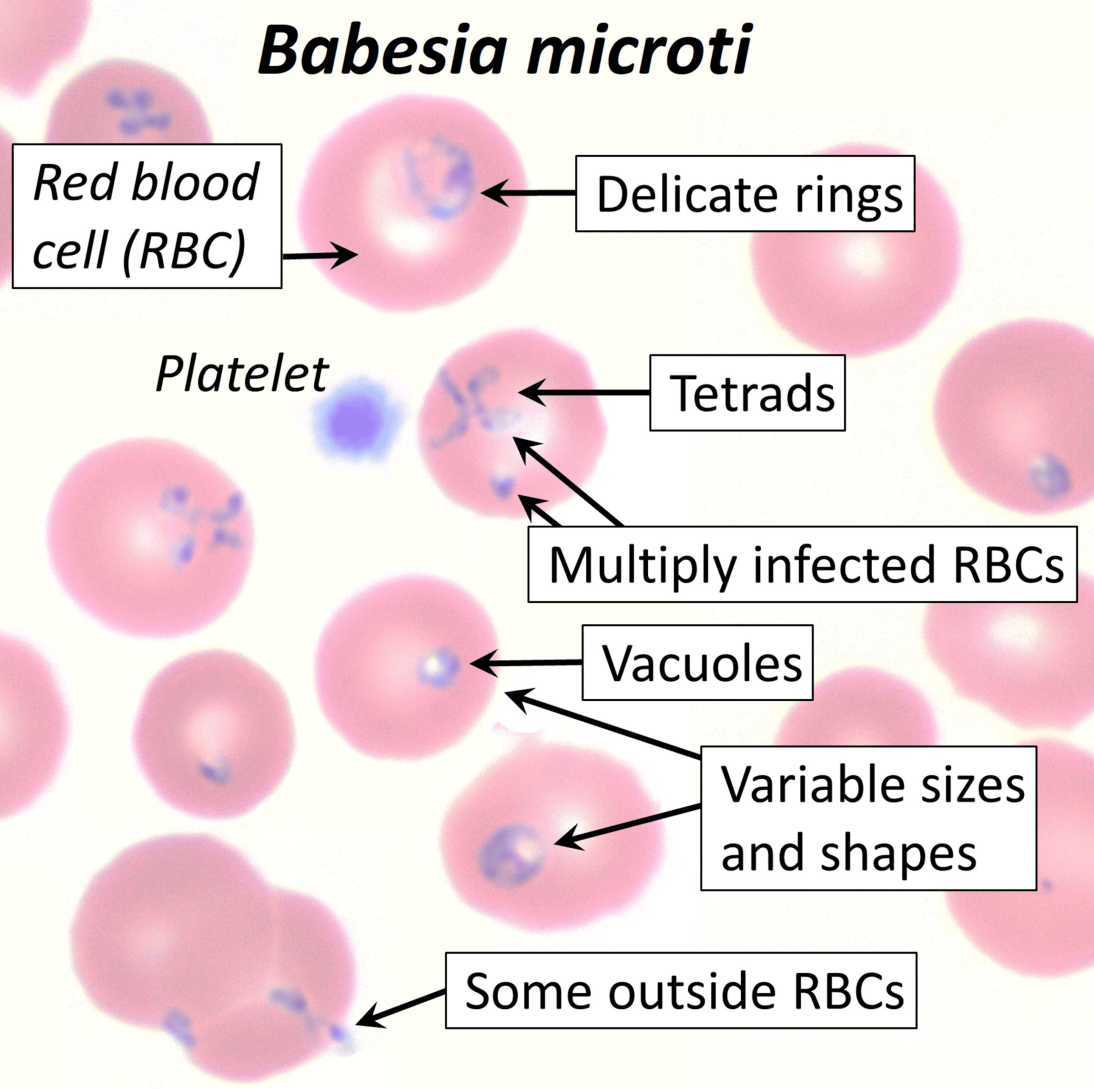
Blood smear of Babesia microti This image shows the various forms in which Babesia species may appear in a blood smear sample. The tetrads form the classic Maltese cross sign correlated with babesiosis. The other forms are non-specific to *Babesia microti*, thus further complicating diagnosis when using a blood smear. From the medical gallery of Mikael Häggström, M.D. Last updated: 2024-07-17. Licensing: Creative Commons CC0 1.0 Universal Public Domain Dedication, used with permission.

Diagnosing babesiosis is challenging due to the nonspecific nature of its symptoms, which overlap with other common febrile illnesses like malaria, Lyme disease, and influenza. This makes it difficult for clinicians to distinguish babesiosis from other diseases, particularly in endemic areas with a high risk of coinfection. The diagnostic approach begins with a high index of suspicion in patients presenting with relevant symptoms and a history of tick exposure. Blood smears remain the gold standard, where Babesia organisms can be identified within red blood cells as the intracellular Maltese Cross sign (Figure 2).

However, blood smears may have low sensitivity, especially when parasitemia is low. Furthermore, the merozoite form may not appear as the classic Maltese cross, thus further complicating diagnosis using blood smears alone. In such cases, molecular diagnostic techniques like PCR testing are invaluable due to their high sensitivity and ability to detect Babesia DNA, even in early or low-parasitemia infections. PCR is particularly useful when blood smears are inconclusive or negative. Serological testing, which detects antibodies to Babesia, is helpful for identifying past infections but is less reliable for diagnosing acute cases since antibody responses take time to develop. Given the potential for co-infections with other tick-borne pathogens, such as Lyme disease and anaplasmosis, a comprehensive diagnostic approach using PCR panels can help differentiate babesiosis from other diseases with overlapping clinical features [5,6].

Treatment for babesiosis typically begins with a combination of atovaquone (750 mg orally twice daily) and azithromycin (500 mg daily), which is recommended for uncomplicated cases due to its effectiveness and low toxicity profile [7,8]. For severe cases, particularly those with parasitemia greater than 4% or in patients who are immunocompromised, a more aggressive regimen of intravenous (IV) clindamycin (600-900 mg every eight hours) combined with quinine (650 mg orally every eight hours) for seven to 10 days is preferred. In life-threatening cases, such as those with parasitemia greater than 10% or severe hemolysis, exchange transfusion or plasmapheresis may be considered to rapidly reduce parasitemia levels [7-9]. Additionally, supportive care such as blood transfusions may be necessary to manage anemia and thrombocytopenia. Regular monitoring of parasitemia levels is essential, especially in severe cases, and may require repeat blood smears or PCR tests to ensure effective resolution of the infection. Long-term follow-up is generally not needed for uncomplicated cases, but patients with severe disease may require additional monitoring for organ damage or persistent symptoms, such as fatigue and malaise, which can last for months in some individuals [7,8].

Preventive measures are the cornerstone of controlling babesiosis, as there is no vaccine available. Preventive strategies focus on reducing tick exposure, especially in endemic areas. These include using tick repellents containing N,N-diethyl-meta-toluamide (DEET) or permethrin on clothing, wearing long sleeves and pants, and performing thorough tick checks after outdoor activities, particularly in areas with a high prevalence of tick-borne diseases. Prompt removal of ticks is essential, as the longer a tick remains attached, the greater the risk of disease transmission. To remove an attached tick, fine-tipped tweezers should be used to grasp the tick as close to the skin as possible, ensuring the entire tick is removed without squeezing, which could increase the likelihood of transmission. These measures, when implemented effectively, can significantly reduce the risk of babesiosis and other tick-borne diseases [10,11].

In this case study, the diagnostic process could have been expedited through several strategies. Given the patient’s history of tick exposure and presenting symptoms such as fever, chills, fatigue, and thrombocytopenia, a higher index of suspicion for tick-borne illnesses, including babesiosis, should have been considered early on. Early diagnostic testing for babesiosis, particularly PCR or a blood smear, would have allowed for more timely identification of the parasite. PCR, with its higher sensitivity for detecting low-parasitemia cases, could have facilitated an earlier diagnosis. Involving an infectious disease specialist sooner would have further expedited the recognition of babesiosis, particularly given the patient’s clinical presentation and potential for severe disease. By prioritizing babesiosis as a possible diagnosis, clinicians could have implemented earlier testing, leading to faster initiation of appropriate therapy. Overall, a more proactive approach to recognizing tick-borne diseases with a corresponding diagnostic workup may have expedited the patient's recovery.

## Conclusions

In conclusion, this case underscores the importance of maintaining a high index of suspicion for tick-borne illnesses, such as babesiosis, in patients with a history of tick exposure and presenting symptoms like fever, chills, body aches, and thrombocytopenia, particularly in endemic regions. Although babesiosis was initially overlooked due to the nonspecific nature of the patient’s symptoms, a timely diagnosis was made following the identification of the characteristic Maltese cross sign on a blood smear. Early involvement of infectious disease specialists and more proactive diagnostic testing, such as PCR for Babesia, could have expedited the diagnosis and treatment, potentially improving the patient's clinical course. This case emphasizes the need for clinicians to consider and test for tick-borne diseases other than Lyme disease when evaluating febrile patients with relevant exposure, and to utilize appropriate diagnostic methods, including blood smear and PCR, to confirm the diagnosis. Furthermore, it highlights the significance of timely intervention and targeted therapy, which, in this case, led to a favorable resolution of the patient’s symptoms and transfer to a higher level of care for continued monitoring.
